# Exploration of Clove Bud (*Syzygium aromaticum*) Essential Oil as a Novel Attractant against *Bactrocera dorsalis* (Hendel) and Its Safety Evaluation

**DOI:** 10.3390/insects13100918

**Published:** 2022-10-09

**Authors:** Zhen-Jie Hu, Jing-Wei Yang, Zi-Han Chen, Cheng Chang, Yu-Pei Ma, Nan Li, Meng Deng, Gen-Lin Mao, Qiang Bao, Shu-Zhen Deng, Huan Liu

**Affiliations:** 1 College of Horticulture and Plant Protection, Henan University of Science and Technology, Luoyang 471023, China; 2 Henan Key Laboratory of Function-Oriented Porous Materials, College of Chemistry and Chemical Engineering, Luoyang Normal University, Luoyang 471934, China; 3 Luoyang Key Laboratory of Organic Functional Molecules, College of Food and Drug, Luoyang Normal University, Luoyang 471934, China; 4 Key Laboratory of South Subtropical Fruit Biology and Genetic Resource Utilization (MOA), Institute of Fruit Tree Research, Guangdong Academy of Agricultural Sciences, Guangzhou 510642, China; 5 Hunan Provincial Tea Research Institute, Hunan Provincial Academy of Agricultural Sciences, Changsha 410125, China

**Keywords:** *Bactrocera dorsalis*, clove bud essential oil, olfactometer bioassay, attractancy, cytotoxicity

## Abstract

**Simple Summary:**

*Bactrocera dorsalis* is among the most economically harmful pests. The lure-and-kill approach is an environmentally friendly and innovative method that offers an opportunity for sustainable surveillance and control of *B. dorsalis*. However, such a strategy requires highly effective pest attractants. In the current study, we compared the attractive activity of twelve flower essential oils against *B. dorsalis* adults via the indoor trapping assay. Meanwhile, we studied the attractive features of clove bud essential oil (CBEO) for males under laboratory conditions. Further, we also investigated CBEO’s attractancy to their natural predator, the ladybirds, and its cytotoxicity against non-target organisms’ cells. In particular, sexually mature males were dramatically attracted to the CBEO. Furthermore, the CBEO exhibited no significant attractancy to ladybirds nor cytotoxicity against normal human and mouse cells. The results revealed that CBEO shows potential for development as an eco-friendly and novel attractant agent for the control of *B. dorsalis*.

**Abstract:**

The oriental fruit fly *Bactrocera dorsalis* (Hendel) is a destructive polyphagous species that targets many economically important fruits and vegetables. The primary control of *B. dorsalis* relies mainly on the use of synthetic chemicals, and excessive use of these chemicals has adverse effects on both the environment and human health. Environmentally friendly management of pests involving plant essential oils is useful for controlling the populations of pests responsible for decreasing the yields and quality of crops. In the present study, we demonstrate that clove bud essential oil (CBEO) is strongly attractive to sexually mature males. Mature males responded to the CBEO differently throughout the day; the strongest response was elicited during the day and decreased at dusk. Virgin and mated mature males did not respond differently to CBEO. No obvious response behaviour to the CBEO was observed in two species of beneficial natural predator ladybirds. In addition, a cytotoxicity assessment demonstrated that CBEO is nontoxic to normal human and mouse cells. Based on our laboratory experiments, CBEO may serve as a promising, sustainable, and environmentally friendly attractant for *B. dorsalis* males; however, field experiments are needed to confirm this hypothesis.

## 1. Introduction

*Bactrocera dorsalis* (Hendel) (Diptera: Tephritidae) is a highly destructive invasive pest throughout the world that is capable of causing extensive damage to a broad range of cultivated and wild fruit and vegetable products [1,2,3]. A serrated ovipositor allows adult females to pierce through the skin of intact ripe fruit and deposit eggs in the interior. The eggs hatch into larvae that tunnel deeper into the fruit as they feed on the fruit pulp, rendering it damaged and unmarketable [2,4]. The application of synthetic insecticides remains an effective method for rapidly suppressing *B. dorsalis* in the field, but it inevitably has detrimental impacts on the environment and human health [2,5,6,7]. Broad-spectrum insecticides may result in the development of insect resistance and deplete populations of natural enemies. In particular, a powerful phytochemical lure, methyl eugenol (ME), has been employed extensively for monitoring, detection, and management of *B. dorsalis* [8,9]. Despite its benefits, ME is associated with risks; it is carcinogenic to humans and is only attractive to male flies, limiting its application for prolonged periods [2,3,10,11]. Therefore, to reduce pesticide application and prevent negative consequences, a more ecologically friendly and novel attractant is urgently required to control *B. dorsalis.*

Plant essential oils (EOs) are natural secondary metabolites derived from aromatic plants and contain complex mixtures of active compounds [12]. In particular, EOs possess antimicrobial, antifungal, antiviral, insecticidal, and herbicidal properties [4,13,14,15]. Studies of the attractant, deterrent, antifeedant, and toxic properties of EOs have been chiefly conducted on numerous phytophagous insects [16,17,18]. For instance, an EO extract from the leaf of *Magnolia citrata* (GiỔi chanh) shows moderately strong attractive power towards males of *Ceratitis capitata* (Wiedemann) (Diptera: Tephritidae) [19]. Moreover, clove oil (*Eugenia caryophyllata* L. Myrtaceae) was studied as a source of attractant to *C. capitata* male flies [20]. *Bactrocera invadens* (Diptera: Tephritidae) males exhibited a significantly positive response to clove essential oils [21]. In addition, a study on olfactory behaviour responses found that clove oil at a 5% concentration is strongly attractive to *Bactrocera zonata* (Saunders) adults [22]. Moreover, Ali et al. [23] reported that clove essential oils exhibited insecticidal activity against *B. zonata* pupa. Thus, in conjunction with other sustainable pest management strategies, EOs can be an effective alternative to conventional plant protection products. However, to date, few studies have investigated the biological activity of EOs, especially clove EO, towards *B. dorsalis*.

A toxicological safety assessment of natural extracts provides scientific evidence of their potential applications. To develop agrochemicals, it is crucial to assess the potential risk of natural extracts for non-target organisms [24,25]. Notably, human or insect cultured cell lines provide a straightforward, ethically compliant, and powerful tool for investigating chemical toxicity [3,26,27]. In the current study, the attractant activity of twelve flower-derived EOs, including clove bud, *Michelia alba* (Magnolianceae), peony, gardenia, peach blossom, jasmine, pear blossom, chrysanthemum, cerasus, *Rosa chinensis* (Yuejihua), *Osmanthus fragrans* Lour., and rose EOs, against mature *B. dorsalis* adults was assessed first. The responsiveness characteristics of *B. dorsalis* males to clove bud essential oil (CBEO) were then evaluated. Further investigations were conducted on the attractancy of CBEO against two natural predators *Coccinella septempunctata* L. and *Harmonia axyridis* (Pallas) (Coleoptera: Coccinellidae). In addition, we further tested the cytotoxicity of CBEO to cells of non-target organisms, including human foetal lung fibroblast 1 (HFL1), human breast cancer (MDA-MB-231), mouse embryonic hepatocytes (BNL-CL.2), and insect ovary (SF-9) cells. This study contributes to the development of a safe, efficient, and sustainable attractant for the control of *B. dorsalis*.

## 2. Materials and Methods

### 2.1. Reagents and Chemicals

Flower essential oils (EOs) under study were purchased from Luxury Bloom Biotech Co., Ltd. (Shanghai, China). Dulbecco’s Modified Eagle Medium (DMEM), a high-sugar medium, was purchased from the HyClone Company (Logan, UT, USA). TNM-FH medium was purchased from Sino Biological Inc. (Beijing, China). Other common chemicals necessary for the experiments were purchased from Sigma Aldrich (Shanghai, China).

### 2.2. Insects Rearing

#### 2.2.1. Bactrocera Dorsalis

*Bactrocera dorsalis* was maintained under laboratory conditions at the Henan University of Science and Technology (HAUST) and reared at 27 ± 1 °C and 75% ± 1% relative humidity with a photoperiod of 14 h:10 h (L:D). *Bactrocera dorsalis* was reared for more than 30 generations. The colony was periodically refreshed with wild flies to maintain genetic diversity. Banana slices were used to collect eggs. Hatching larvae were reared on an artificial diet based on that used in a previous report [11,28]. The adult flies were reared in mesh cages (30 cm × 30 cm × 30 cm) and fed on an artificial diet consisting of dry sugar:yeast extract (1:1, *w*/*w*) and water [3,27].

#### 2.2.2. Ladybirds

Adults of the natural predators *Coccinella septempunctata* L. (Coleoptera: Coccinellidae) (seven-spotted ladybeetle) and *Harmonia axyridis* Pallas (Coleoptera: Coccinellidae) were collected from the research experimental field of HAUST. Both larval and adult ladybirds were fed with green peach aphids and reared under laboratory conditions of 25 ± 1 °C, 65% ± 5% relative humidity, and a 12 h light regime [29,30]. Cotton wool soaked in water served as a source of water. Larvae and adult ladybirds (3 days after eclosion) were chosen for tests.

### 2.3. Cell Culture

Cells of human foetal lung fibroblast 1 (HFL1), human breast cancer cell (MDA-MB-231), and mouse embryonic hepatocytes (BNL-CL.2) were cultivated in DMEM supplemented with 100 µg/mL of streptomycin, 100 U/mL of penicillin, and 10% foetal bovine serum (FBS) in an incubator with a humidified air atmosphere of 5% CO2 at 37 °C. The MDA-MB-231, HFL1, and BNL-CL.2 cells were purchased from Procell Life Science and Technology Co., Ltd. (Wuhan, China). *Spodoptera frugiperda* (Lepidoptera: Noctuidae) vary cells (SF-9) were kindly donated by the Fruit Tree Research Institute, Guangdong Academy of Agricultural Sciences (Guangzhou, China). The SF-9 cells were maintained in TNM-FH medium supplemented with 1% penicillin and 10% FBS and grown in a humidified incubator at 28 °C. All tests were conducted with exponentially growing cells [3,27].

### 2.4. Responsiveness of B. Dorsalis Flies to Flower-Derived EOs in an Indoor Trapping Assay

Based on recently reported methods [3,27], we compared the taxes of *B. dorsalis* mature flies to the flower-derived EOs, including clove bud, *Michelia alba*, peony, gardenia, peach blossom, jasmine, pear blossom, chrysanthemum, cerasus, *Rosa chinensis*, *Osmanthus fragrans*, and rose EOs. Initially, EO solutions were prepared using 5% Tween-80, with final concentrations of 50, 100, 200, 400, 800, and 1000 µL/mL. Subsequently, 40 mature flies (16 days old, male:female = 1:1) were randomly selected as subjects and placed in a cubical screen cage (35 cm per side). After approximately 30 min, a fly trap was placed inside each cage. The fly trap was constructed from 150 mL conical flasks on which a silicone top containing a 15 mL centrifuge tube (cut for 3.5 cm) was securely placed. Each trap contained a 1.5 mL tube with a cotton wick with 100 µL of the trial concentration of the EOs. In the control groups, 5% Tween-80 was used in the traps. Traps were removed after 2 h, and trapped flies were counted. Experiments were biologically independently replicated five times. Experiments were conducted from 09:00 to 11:00. During the experimental period, the ambient temperature and relative humidity were controlled at 27 ± 2 °C and 75% ± 5%, respectively. The responsiveness ratio of *B. dorsalis* flies to the flower-derived EOs was calculated with the following formula:

$$Response\;rate\;\left(\%\right)=\frac{No.\;of\;flies\;trapped\;in\;treatment-No.\;of\;flies\;trapped\;in\;control\;}{No.\;of\;test\;flies}\times 100\%$$

### 2.5. Effect of Age, Daily Rhythm, and Physiological Condition on Male Taxis to CBEO

For this experiment, 40 male flies aged 2, 4, 6, 8, 12, 16, 20, 24, 28, and 32 days were selected randomly to determine the influence of age on CBEO sensitivity in male flies. A trapping assay was conducted as previously described. Sixteen-day-old male flies were assessed in three separate experiments at 09:00 (morning), 13:00 (early afternoon), and 17:00 (near dusk) to assess whether the responses of mature males to CBEO varied throughout the day. Furthermore, to determine whether mating status affects the responsiveness of males to CBEO, a comparison between virgin and mated males (16 days old) was conducted at 09:00, 13:00, and 17:00. Finally, the responses of starveling or thirsty males (16 days old) to CBEO were assayed to determine whether the responses of mature males to CBEO vary according to their physiological condition. Each bioassay was repeated five times.

### 2.6. Attractancy of CBEO to Natural Predator Ladybirds

In this experiment, the responses of larval (1st instar, 2nd instar, 3rd instar, and 4th instar) and adult (3 days old) ladybirds to CBEO were investigated using a Y-tube olfactometer apparatus (20 cm upstream arm, 25 cm common arm, 3 cm internal diameter, and 60° branching angle). Bioassays were conducted in accordance with a previously published protocol [31,32,33]. Treatment with CBEO at 400 µL/mL was applied as a test stimulus. An olfactometer arm was fitted with a filter paper strip containing 10 µL of an odour stimulus solution. The opposite arm, which served as the control, was coated with 5% Tween-80 (10 µL) on filter paper. Each olfactometer arm was pumped with 100 mL of purified air every minute. The Y-tubes were enclosed in a white fabric box to reduce differences in light intensity.

After turning on the air pump for 5 min, ladybirds pre-starved for 4 h were used in the olfactory experiments. Each group of 10 samples was selected randomly and placed into the common arm tube simultaneously. Each bioassay test was replicated five times. After acclimatization for 2 min, participants were allowed to move freely in the Y-tube for 8 min. It was deemed an effective choice when the individual entered one arm tube, passed one-third of its length, and remained for 30 s. Non-responders were those that did not meet the abovementioned criteria. After testing 10 ladybirds, odour sources were interchanged, and the positions of the stimuli were rotated to avoid any influence of unforeseen asymmetries in the setup. Following each test, acetone and distilled water were used to clean the Y-tube testing devices. Each ladybird was tested only once. All bioassays were conducted daily from 09:00 to 17:00 under 70% ± 5% relative humidity and 27 ± 1 °C. The following formula was used for the calculation of ladybird response ratios to CBEO [3]:

$$Response\;ratios\;\left(\%\right)=\frac{Number\;of\;responding\;ladybirds\;for\;one\;arm\;}{Total\;number\;of\;responding\;ladybirds}\times 100\%$$

### 2.7. Cytotoxicity Test

MTT assays were used to test the cellular toxicity of CBEO against MDA-MB-231, HFL1, SF-9, and BNL-CL.2 cells using a previously described method [3,34,35]. Briefly, cells (1 × 10^5^ cells/well) were seeded in a 96-well plate in a final volume of 100 µL/well. After 24 h of priming, cells were treated with different concentrations of CBEO solutions in dimethyl sulfoxide (DMSO) (15.625, 31.25, 62.5, 125, 250, 500, and 1000 μg/mL) and incubated for 24 h. Controls received fresh media containing 0.5% DMSO solution. After 24 h, the cell viability was investigated by adding 10 µL of MTT solution (5 mg/mL) to the medium. After incubation for a further 4 h, the supernatant was removed and 100 µL DMSO was added to dissolve formazan crystals. Subsequently, the optical density (OD value) of the mixture was measured at a wavelength of 570 nm. The experiment was conducted independently in triplicate. Cell viability was calculated with the following formula:

$$Cell\;viability\;\left(\%\right)=\frac{OD_{treatment}\;\;}{OD_{control}}\times 100\%$$

### 2.8. Data Analysis

Data analysis was conducted using SAS v9.20 (SAS Institute, Inc., Cary, NC, USA). GraphPad Prism 8.0 software was used to draw the figures. Each variable was expressed as the mean and standard error (SE). The statistical significance of differences between experimental and control groups was assessed using one-way ANOVA and Dunnett’s multiple comparison post hoc test. Ladybird distribution in the Y-tube olfactometer bioassays was compared by chi-square analysis. Unresponsive ladybirds were excluded from the analyses. Prior to analysis, the percentage data were transformed with arcsine. A *p*-value < 0.05 was considered to be statistically significant.

## 3. Results

### 3.1. Behavioural Response of B. Dorsalis Mature Flies to Flower-Derived EOs

**Figure 1:**
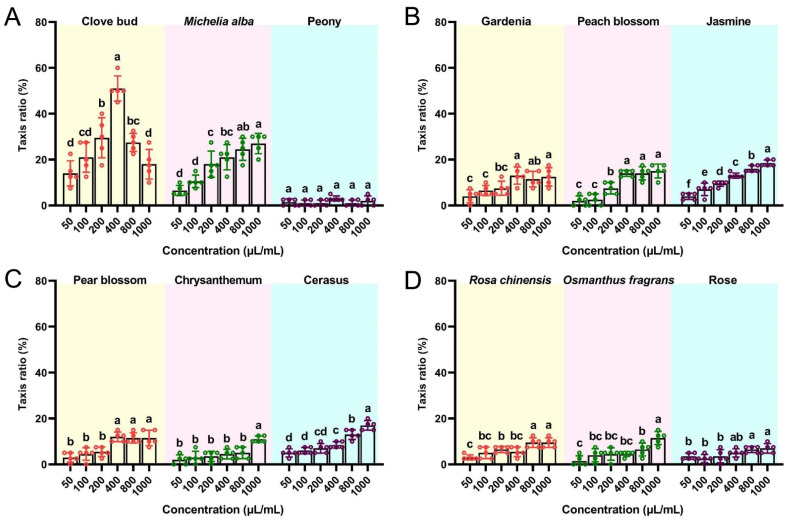
Behavioural response of *Bactrocera dorsalis* mature flies to flower-derived essential oils (EOs). Attractant activity to mature flies was assessed using a trapping method for different concentrations of (**A**) clove bud, *Michelia alba*, and peony EOs; (**B**) gardenia, peach blossom, and jasmine EOs; (**C**) pear blossom, chrysanthemum, and cerasus EOs; and (**D**) *Rosa chinensis*, *Osmanthus fragrans*, and rose EOs. Each bar represents the mean ± SEM of five replicates. Different lowercase letters indicate significant differences (ANOVA, *p* < 0.05).

We compared the attractant activities of different doses of flower-derived EOs to mature flies by performing an indoor trapping assay (Figure 1). The EOs at different concentrations exhibited different levels of attractiveness toward *B. dorsalis* adults. In comparison with the other flower-derived EOs, the CBEO was more effective at attracting mature flies (Figure 1A). At concentrations ranging from 50 to 400 µL/mL, the CBEO showed significant enhancement of attractiveness. In contrast to the other concentrations, the CBEO at 400 µL/mL exhibited the highest attractant activity (51.00% ± 2.45%) (*F* = 32.19; *df* = 5; *p* < 0.0001). The trapping frequency of flies attracted to the *Michelia alba* EO was highest at 1000 µL/mL (27.00% ± 2.00%) (*F* = 19.64; *df* = 5; *p* < 0.0001), which was significantly lower than that of the CBEO at 400 µL/mL (*t* = 5.58; *df* = 4; *p* = 0.0051).

### 3.2. Behavioural Activities of Male and Female Flies in Response to Flower-Derived EOs

**Figure 2:**
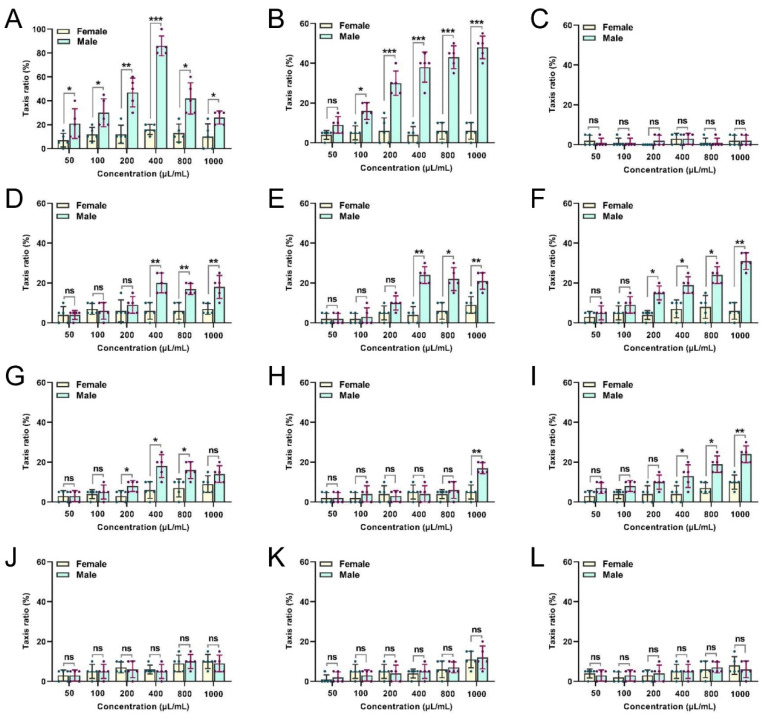
Comparative responsiveness of male and female flies to different concentrations of flower-derived essential oils (EOs). Responsiveness to (**A**) clove bud, (**B**) *Michelia alba*, (**C**) peony, (**D**) gardenia, (**E**) peach blossom, (**F**) jasmine, (**G**) pear blossom, (**H**) chrysanthemum, (**I**) cerasus, (**J**) *Rosa chinensis*, (**K**) *Osmanthus fragrans*, and (**L**) rose EOs was tested. Each bar represents the mean ± SEM of five replicates. Asterisks indicate a significant difference between the sexes (*t*-tests, *p* < 0.05). * *p* < 0.05, ** *p* < 0.01, *** *p* < 0.001, ns = not significant (*p* > 0.05).

Several of the tested EOs had clear levels of attractiveness to *B. dorsalis* males and females at various concentrations (Figure 2). Females and males were not attracted by the peony EOs source (Figure 2C). Notably, female and male flies showed a significant preference for CBEO (Figure 2A). The highest trapping rate of females by the CBEO at 400 µL/mL was 16.00% ± 1.87%, which is significantly lower than the attractiveness of the same concentration of CBEO to males (86.00% ± 3.67%) (*t* = 22.14; *df* = 4; *p* < 0.0001). Compared with females, males were more likely to be trapped by the CBEO. In addition, the trapping rate of males in response to the *Michelia alba* EO at 1000 µL/mL was the highest (48.00% ± 2.55%) (Figure 2B).

### 3.3. Attractiveness Characteristics of CBEO to Male Flies

**Figure 3:**
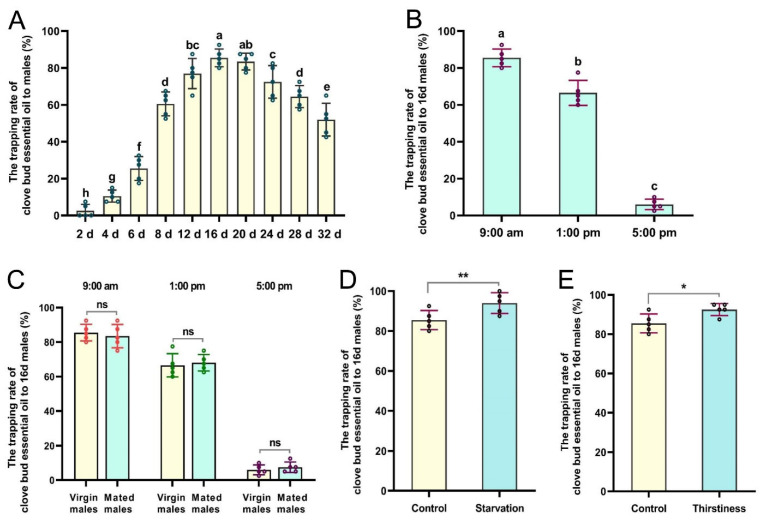
Responsiveness of *Bactrocera dorsalis* male flies to clove bud essential oil (CBEO): (**A**) Age-related response of males to CBEO. (**B**) Diurnal pattern of mature virgin male responsiveness to CBEO. (**C**) Diurnal pattern of virgin and mated male responsiveness to CBEO. (**D**,**E**) Response of starved or thirsty males to CBEO. Each bar represents the mean ± SEM of five replicates. Different lowercase letters indicate significant differences (ANOVA, *p* < 0.05). Asterisks denote a significant difference between treatments (*t*-tests, *p* < 0.05). * *p* < 0.05, ** *p* < 0.01, ns = not significant (*p* > 0.05).

Significant variations in the age-related trappings of CBEO were evident among male flies (Figure 3A). The taxes presented by young males (2- and 4-day-old) to the CBEO were the lowest. In particular, the mean response rates of 12-, 16-, and 20-day-old male flies to the CBEO were 77.00% ± 3.66%, 85.50% ± 2.15%, and 83.50% ± 2.03%, respectively, which were significantly higher than that of any other age categories (*F* = 128.88; *df* = 9; *p* < 0.0001). Even at 28 and 32 days of age, males were still fairly highly attracted to the CBEO. Interestingly, there was a noticeable difference between males throughout the day in their response to the CBEO. In the morning (09:00) and early afternoon (13:00), sexually mature males responded most strongly to the CBEO, but their response declined dramatically at dusk (17:00) (*F* = 356.60; *df* = 2; *p* < 0.0001) (Figure 3B). Moreover, neither virgin nor mated mature males responded significantly to the CBEO (*p* > 0.05) (Figure 3C). In addition, compared with the control groups, male flies suffering from starvation or thirst showed a relatively significant preference for the CBEO (*t* = 6.67, *df* = 4, *p* = 0.0026; *t* = 2.89, *df* = 4, *p* = 0.0447) (Figure 3D,E).

### 3.4. Two-Choice Assays of C. Septempunctata and H. Axyridis in a Y-Tube Olfactometer

**Figure 4:**
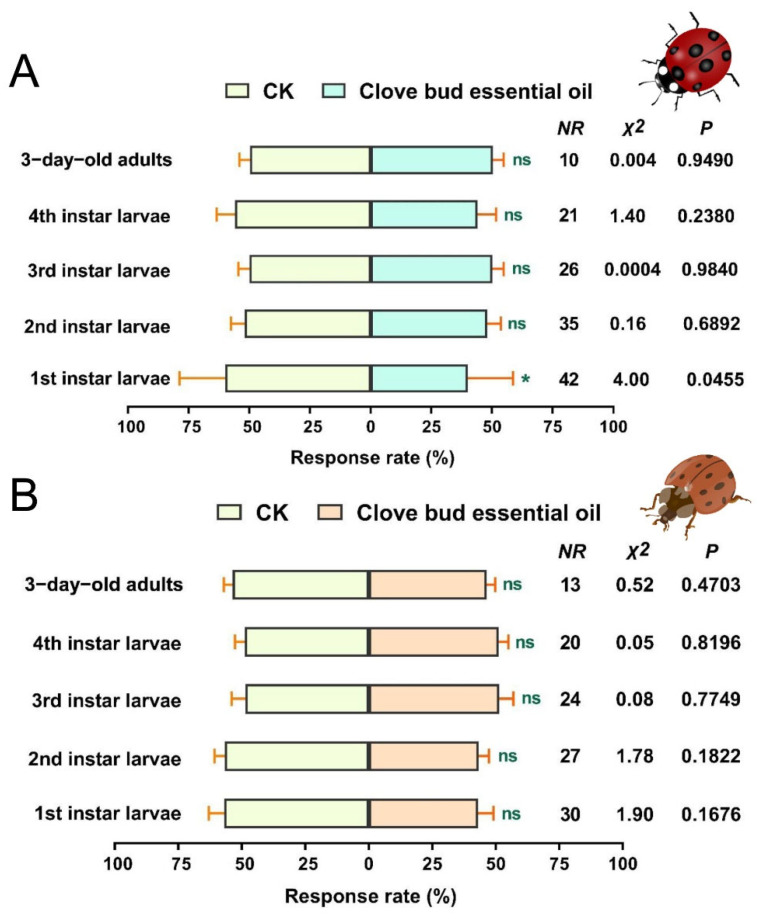
Preferences of *Coccinella septempunctata* and *Harmonia axyridis* to CBEO were assessed with a Y-tube olfactometer bioassay. (**A**) Behavioural response of *C. septempunctata* to 400 µL/mL of CBEO. (**B**) Behavioural response of *H. axyridis* to 400 µL /mL of CBEO. CK represents 5% Tween-80 treatment. NR (no response) indicates the number of ladybirds that failed to respond within the allotted timeframe. Each bar represents the mean ± SEM of five replicates. Asterisks represent a significant difference in preference (chi-square test, *p* < 0.05). * *p* < 0.05, ns = not significant (*p* > 0.05).

The responses of the tested *C. septempunctata* and *H. axyridis* in the Y-tube olfactometer for the CBEO at 400 µL/mL are shown in Figure 4A, B. In the dual-choice olfactometer assay, more *C. septempunctata* first-instar larvae were attracted by the 5% Tween-80 compared with the 400 µL/mL of CBEO (*χ^2^* = 4.00, *df* = 1, *p* = 0.0455). However, second-instar, third-instar, and fourth-instar larvae as well as 3-day-old adults of *C. septempunctata* showed no significant preference for the CBEO compared with the control (*p* > 0.05) (Figure 4A). Similarly, *H. axyridis* larvae and adults showed no obvious preference between the CBEO and the control (*p* > 0.05) (Figure 4B). Overall, *C. septempunctata* and *H. axyridis* were unable to recognize the odours emitted by CBEO.

### 3.5. Cytotoxicity of CBEO against SF-9, BNL-CL.2, HFL1, and MDA-MB-231 Cells

**Figure 5:**
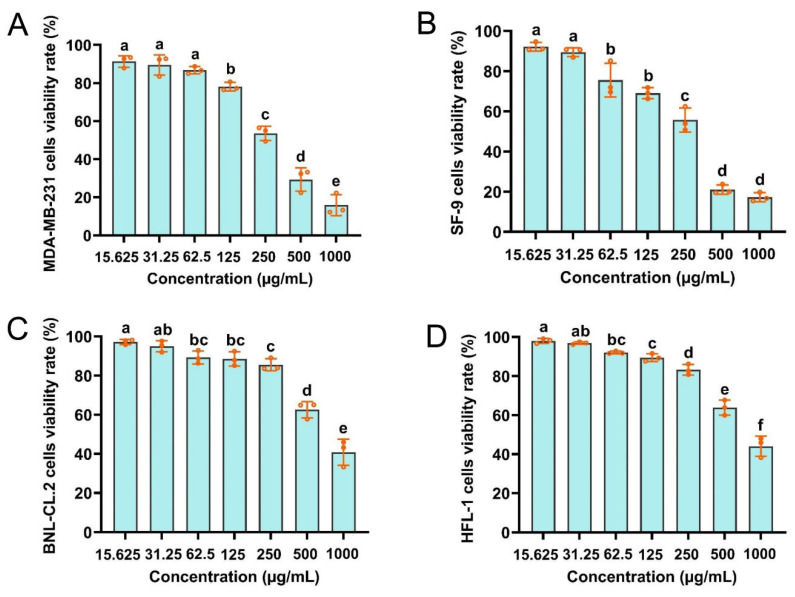
Clove bud essential oil (CBEO) affects the viability of SF-9, BNL-CL.2, HFL1, and MDA-MB-231 cells. Cellular toxicity of CBEO to (**A**) MDA-MB-231 cells, (**B**) SF-9 cells, (**C**) BNL-CL.2 cells, and (**D**) HFL1 cells. Each bar represents the mean ± SEM of three replicates. Different lowercase letters denote significant differences (ANOVA, *p* < 0.05).

MTT assays were performed to determine the cytotoxicity of CBEO against SF-9, BNL-CL.2, HFL1, and MDA-MB-231 cells. The trend tests consistently showed a positive correlation between the CBEO concentration and the inhibitory effect (Figure 5). As evidenced by these tests, CBEO at concentrations ranging from 125 to 1000 g/mL significantly inhibited the viability of MDA-MB-231 cells (*F* = 149.07; *df* = 6; *p* < 0.0001) (Figure 5A). Likewise, CBEO at concentrations ranging from 62.5 to 1000 μg/mL significantly inhibited SF-9 cell viability (*F* = 174.78; *df* = 6; *p* < 0.0001) (Figure 5B). The *IC_50_* values of CBEO on MDA-MB-231 and SF-9 cells were 257.6722 μg/mL and 212.5499 μg/mL, respectively (Table 1). Notably, the CBEO exhibited no significant cellular toxicity on HFL1 and BNL-CL.2 cells (Figure 5C,D). Only exposure to high concentrations (1000 g/mL) of CBEO for 24 h significantly inhibited the viability of the HFL1 (*F* = 135.54; *df* = 6; *p* < 0.0001) and BNL-CL.2 cells (*F* = 86.47; *df* = 6; *p* < 0.0001). *IC_50_* values of 1053.3570 μg/mL and 1060.4790 μg/mL were observed for the CBEO on HFL1 and BNL-CL.2 cells, respectively (Table 1). Consequently, CBEO was demonstrated to be non-cytotoxic to normal human and mouse cells.

## 4. Discussion

The oriental fruit fly (*Bactrocera dorsalis*) is a globally economically destructive pest that attacks various fruit and vegetable products. At present, the lure-and-kill technology is an important and effective strategy for rapidly controlling *B. dorsalis* [2,3]. The exploitation of environmentally friendly and effective attractants for the early detection and suppression of *B. dorsalis* populations is critical for managing this pest and reducing crop losses. In particular, a search is required for safe and biorational attractant agents from natural sources that could be useful in future *B. dorsalis* management strategies. In the current study, an indoor trapping trial revealed that CBEO was the most highly attractive EO to *B. dorsalis* mature adults. Notably, CBEO was significantly more attractive to males than to females. A dramatic attraction to the CBEO prior to dusk was observed among sexually mature virgin and mated males. In addition, Y-tube bioassays and cellular toxicity tests revealed that CBEO was safe for non-target organisms. In summary, these results provide timely, valuable information on the environmental friendliness of CBEO and promote its development as a promising green bait for detecting and controlling *B. dorsalis*.

Plant extracts have been considered for the development of new and less toxic control methods due to their low toxicity and low environmental impact [36]. The biological activity of EOs strictly depends on their chemical compositions, which vary according to the plant parts used, drying method, extraction technique, plant age, plant phenological stage, harvesting season, soil composition, and environmental conditions in which the plant grows [4,13]. The present research revealed that certain EOs were effective as attractants of *B. dorsalis* to some degree in an indoor trapping experiment. The EO from *Michelia alba* (Magnolianceae) was slightly attractive to *B. dorsalis* males. Among all examined EOs, the CBEO had the highest attractant efficacy for *B. dorsalis* males.

The evergreen tree *Syzygium aromaticum* (L.) Merr. & L.M.Perry, belonging to the Myrtaceae family, produces aromatic flower buds commonly known as cloves [37]. Previous publications confirmed that clove oil extract shows potential attractant activity against males of two species of fruit flies: *C. capitata* and *Bactrocera zonata* (Saunders) (Diptera: Tephritidae). In contrast, female *C. capitata* and *B. zonata* do not show a positive response to the clove oil extract [36]. Furthermore, as reported by Bulawan et al. [38], *B. dorsalis* adults were captured most frequently in clove leaf extract treatments. CBEO is extracted from dried flower buds of the clove tree [39]. The present findings are in agreement with those of Moustafa et al. [36] and Bulawan et al. [38]. Of particular importance is the fact that the attractant efficiency of an EO is associated with its composition. The major constituents identified in CBEO are eugenol, β-caryophyllene, *O*-allylguaiacol, α-humulene, β-thujaplicin, caryophyllene oxide, and lesser amounts of other components, such as benzyl alcohol, 2-heptanol, 2-nonanone, 2-nonanol, 2-undecanone, and carvone [40,41,42]. The compound β-caryophyllene was demonstrated to be a more specific and potent male lure for *B. correcta* and *C. capitata* [43,44]. Moreover, it has been demonstrated that the relatively low concentration of β-caryophyllene attracts *B. dorsalis* gravid females [45]. A previous study also revealed that α-humulene is clearly attractive to males of *C. capitata* [43]. Additionally, 2-nonanone and 2-undecanone have been reported to stimulate a strong antennal response and a positive oviposition response on *C. capitata*, acting as an attractant and oviposition stimulant [46]. Although the constituents of clove bud oils can differ, it is highly likely that eugenol is the primary component of clove EO, according to all reports [37,39,40,41,47,48,49]. Although the attractiveness of eugenol to *B. dorsalis* has not been reported, several of its analogous compounds have been characterized as male attractants and male sex pheromones of *B. dorsalis* [50]. In addition, eugenol has a broad range of pharmacological effects, including local anaesthetic, analgesic, antimicrobial, anti-inflammatory, antitumor, and hair-growing properties [51,52,53]. Moreover, eugenol has been generally recognized as safe (GRAS) as a direct human food ingredient [54].

Notably, the positive response of *B. dorsalis* males to CBEO increased with age. Sexually immature males did not respond to CBEO as early as 2 and 4 days after emergence (DAE). Biologically, male adults become sexually mature at approximately 10 DAE [27,28]. It was interesting to observe that the attraction of the male flies to the CBEO peaked during the period from 12 to 24 DAE, which coincided with the sexual maturity of the flies. Immature males of *B. dorsalis* spend most of the day hunting for protein and sugar to supplement their nutritional requirements, whereas mature males respond favourably to sex pheromones or parapheromones [55,56]. These results indicate that CBEO plays a more important role among *B. dorsalis* males for chemical and ecological purposes than for nutritional functions. The attractiveness of a volatile compound is not solely determined by its chemical properties and changes with the physiological state of an insect [3,11,57]. Under starvation and thirst stress, *B. dorsalis* males became significantly more responsive to CBEO. Moreover, the responsiveness of sexually mature males to CBEO fluctuated during the day: they responded most strongly in the morning and afternoon and responded less markedly near dusk. As a dusk-mating species, its courtship behaviour is restricted to the period from 17:00 to 19:00 [11]. In this context, during the courtship phase, *B. dorsalis* males may transiently cease responding to the CBEO in favour of female sex pheromones. In addition, *B. dorsalis* adults are known to perform multiple mating [3,58]. The behavioural responses to CBEO were not significantly different between virgin and mated males. Undoubtedly, knowledge of the characteristics of male flies’ responsiveness to CBEO is vital in the planning and monitoring of *B. dorsalis* control programmes.

The increasing consumption of natural extracts has raised concerns about their safety. To use natural extracts effectively, an assessment of their safety is necessary [59,60]. In this context, prior to endorsing CBEO as a novel male attractant for *B. dorsalis* control, certain issues regarding its ecotoxicological safety should be considered. The ladybirds *C. septempunctata* and *H. axyridis* are important and beneficial natural predators. Although they are not predators of *B. dorsalis*, they have been widely used as the main biocontrol agents against a variety of aphids in greenhouses and farmland [30,61,62,63]. These beetles are recognized as widely distributed in agricultural and natural habitats around the world. However, these predators are negatively affected by unintended and indiscriminate exposure to synthetic insecticides [30,64]. Specifically, in the current evaluation, the CBEO possessed no significant attractancy for larvae and adults of *C. septempunctata* and *H. axyridis.* A previous study revealed that clove EO has no acute toxicity to *Coleomegilla maculata* (De Geer) (Coleoptera: Coccinellidae), a non-target ladybeetle. In addition, *C. maculata* exposed to clove EO exhibited unaffected locomotion and aphid predation abilities [65]. Furthermore, clove oil is reportedly non-toxic to fish [39].

Notably, cultivable cell-based systems present rapid in vitro assays for assessment of the potential toxicity and risk of xenobiotic chemicals [3,26,27,66]. In addition to being accurate and effective, these systems use no animals or humans, which eliminates any ethical dilemmas [27,67]. Based on the present cytotoxicity investigation, CBEO was indicated to be highly cytotoxic to human cancer cells (MDA-MB-231) and insect cells (SF-9) but relatively showed low or no toxicity to normal human and mouse cells (HFL1 and BNL-CL.2). Therefore, CBEO is likely to possess anticancer and insecticidal properties. Coincidentally, clove EO shows anticarcinogenic, antimutagenic, and insecticidal potential [39,42,47,68,69]. These observations accord well with the present results. Moreover, clove buds and clove oil have been approved as generally safe by the U.S. Food and Drug Administration [70]. In addition, the U.S. Environmental Protection Agency has classified these as minimum-risk pesticides. Overall, the present cytotoxicity study further reveals that CBEO can be regarded as nontoxic and exhibits a good environmental safety performance.

## 5. Conclusions

In short, the current results show that CBEO may be developed as a safe and potent lure for *B. dorsalis* male flies, which may help improve and optimize current trapping control techniques. Though the premise is promising, the use of CBEO as bait in attract-and-kill programmes in this field is still poorly investigated and implemented. Moreover, the bioactivity of CBEO on other non-target organisms requires further study.

## Figures and Tables

**Table 1:** Assessment of cytotoxicity of CBEO against SF-9, BNL-CL.2, HFL1, and MDA-MB-231 cells for 24 h.

Cell Line	Toxicity Regression Equation	Correlation Coefficient	*IC_50_* (µg/mL)
SF-9	*y* = 1.7605 + 1.3919*x*	0.9793	212.5499
MDA-MB-231	*y* = 1.6595 + 1.3855*x*	0.9658	257.6722
HFL1	*y* = 1.3911 + 1.1940*x*	0.9835	1053.3570
BNL-CL.2	*y* = 1.6807 + 1.097*x*	0.9596	1060.4790

## Data Availability

All data sets presented in this study are included in the article and can be provided by the authors upon reasonable request.

## References

[1] Zheng W.W., Peng W., Zhu C.P., Zhang Q., Saccone G., Zhang H.Y. (2013). Identification and expression profile analysis of odorant binding proteins in the Oriental fruit fly *Bactrocera dorsalis*. Int. J. Mol. Sci..

[2] Liu H., Zhang D.J., Xu Y.J., Wang L., Cheng D.F., Qi Y.X., Zeng L., Lu Y.Y. (2019). The invasion, expansion and prevention of *Bactrocera dorsalis* (Hendel) in China. J. Integr. Agric..

[3] Liu H., Wang D.D., Wan L., Hu Z.Y., He T.T., Wang J.B., Deng S.Z., Wang X.S. (2022). Assessment of attractancy and safeness of (E)-coniferyl alcohol for management of female adults of Oriental fruit fly, *Bactrocera dorsalis* (Hendel). Pest. Manag. Sci..

[4] Zeni V., Benelli G., Campolo O., Giunti G., Palmeri V., Maggi F., Rizzo R., Lo Verde G., Lucchi A., Canale A. (2021). Toxics or lures? Biological and behavioral effects of plant essential oils on tephritidae fruit flies. Molecules.

[5] Jin T., Zeng L., Lin Y.Y., Lu Y.Y., Liang G.W. (2011). Insecticide resistance of the oriental fruit fly, *Bactrocera dorsalis* (Hendel) (Diptera: Tephritidae), in mainland China. Pest Manag. Sci..

[6] Vontas J., Hernández-Crespo P., Margaritopoulos J.T., Ortego F., Feng H.T., Mathiopoulos K.D., Hsu J.C. (2011). Insecticide resistance in tephritid flies. Pestic. Biochem. Physiol..

[7] Yoon M.Y., Cha B., Kim J.C. (2013). Recent trends in studies on botanical fungicides in agriculture. Plant. Pathol. J..

[8] Shelly T.E. (2017). Zingerone and the mating success and field attraction of male melon flies (Diptera: Tephritidae). J. Asia-Pac. Entomol..

[9] Wee S.L., Abdul Munir M.Z., Hee A.K.W. (2018). Attraction and consumption of methyl eugenol by male *Bactrocera umbrosa* Fabricius (Diptera: Tephritidae) promotes conspecific sexual communication and mating performance. Bull. Entomol. Res..

[10] Zheng W., Zhu C., Peng T., Zhang H. (2012). Odorant receptor co-receptor *Orco* is upregulated by methyl eugenol in male *Bactrocera dorsalis* (Diptera: Tephritidae). J. Insect. Physiol..

[11] Liu H., Chen Z.S., Zhang D.J., Lu Y.Y. (2018). *BdorOR88a* modulates the responsiveness to methyl eugenol in mature males of *Bactrocera dorsalis*. Front. Physiol..

[12] Gaire S., Zheng W., Scharf M.E., Gondhalekar A.D. (2021). Plant essential oil constituents enhance deltamethrin toxicity in a resistant population of bed bugs (*Cimex lectularius* L.) by inhibiting cytochrome P450 enzymes. Pestic. Biochem. Physiol..

[13] Said-Al Ahl H.A., Hikal W.M., Tkachenko K.G. (2017). Essential oils with potential as insecticidal agents: A review. Int. J. Environ. Plan. Manag..

[14] Sumalan R.M., Alexa E., Popescu I., Negrea M., Radulov I., Obistioiu D., Cocan I. (2019). Exploring ecological alternatives for crop protection using *Coriandrum sativum* essential oil. Molecules.

[15] Isman M.B. (2020). Commercial development of plant essential oils and their constituents as active ingredients in bioinsecticides. Phytochem. Rev..

[16] Benelli G., Pavela R. (2018). Beyond mosquitoes-essential oil toxicity and repellency against bloodsucking insects. Ind. Crop. Prod..

[17] Li M.X., Ma Y.P., Zhang H.X., Sun H.Z., Su H.H., Pei S.J., Du Z.Z. (2020). Repellent, larvicidal and adulticidal activities of essential oil from Dai medicinal plant *Zingiber cassumunar* against *Aedes albopictus*. Plant Divers..

[18] Liu H., Guo S.S., Lu L., Li D., Liang J., Huang Z.H., Zhou Y.M., Zhang W.J., Du S. (2021). Essential oil from *Artemisia annua* aerial parts: Composition and repellent activity against two storage pests. Nat. Prod. Res..

[19] Luu-Dam N.A., Tabanca N., Estep A.S., Nguyen D.H., Kendra P.E. (2021). Insecticidal and attractant activities of *Magnolia citrata* leaf essential oil against two major pests from Diptera: *Aedes aegypti* (Culicidae) and *Ceratitis capitata* (Tephritidae). Molecules.

[20] El-Kareim A., Shanab L.M., El-Naggar M.E., Ghanim N.M.A. (2009). The efficacy of some volatile oil extracts as olfactory stimuli to the fruit flies, *Bactrocera zonata* (Saunders) and *Ceratitis capitata* (Wiedemann) (Diptera: Tephritidae). J. Plant Prot. Pathol..

[21] Calvert M.C., Gucker L. (2014). The invading fly: Innovative pest management solutions for control of *Bactrocera invadens* in Pemba, Zanzibar. Indep. Study Proj. Collect..

[22] El-Banna B.S.M., Gab Alla M.A.A. (2021). Evaluation of some fruits extracts and oils to attracted peach fruit fly, *Bactrocera zonata* (Saunders) by simple olfactometer design under laboratory conditions. J. Plant Prot. Pathol..

[23] Ali M.A. (2018). Toxicity of certain plant oils on pupil stage of the peach fruit fly, *B. zonata* (Sunders) (Tephritidae: Diptera). Adv. Plants Agric. Res..

[24] Wang Z., Brooks B.W., Zeng E.Y., You J. (2019). Comparative mammalian hazards of neonicotinoid insecticides among exposure durations. Environ. Int..

[25] Zhu Q., Yang Y., Lao Z., Zhong Y., Zhang K., Zhao S. (2020). Acute and chronic toxicity of deltamethrin, permethrin, and dihaloacetylated heterocyclic pyrethroids in mice. Pest. Manag. Sci..

[26] Song U., Kim J. (2020). Assessment of the potential risk of 1,2-hexanediol using phytotoxicity and cytotoxicity testing. Ecotoxicol. Environ. Saf..

[27] Deng S.Z., Li X.Y., Wang Z.M., Wang J.B., Han D.Y., Fan J.H., Zhao Q., Liu H., Wang X.S. (2021). Assessment of 2-allyl-4,5-dimethoxyphenol safety and attractiveness to mature males of *Bactrocera dorsalis* (Hendel). Ecotoxicol. Environ. Saf..

[28] Liu H., Zhao X.F., Fu L., Han Y.Y., Chen J., Lu Y.Y. (2017). *BdorOBP2* plays an indispensable role in the perception of methyl eugenol by mature males of *Bactrocera dorsalis* (Hendel). Sci. Rep..

[29] Zhao Y., Yun Y., Peng Y. (2020). *Bacillus thuringiensis* protein Vip3Aa does not harm the predator *Propylea japonica*: A toxicological, histopathological, biochemical and molecular analysis. Ecotoxicol. Environ. Saf..

[30] Qin D., Liu B., Zhang P., Zheng Q., Luo P., Ye C., Zhao W., Zhang Z. (2021). Treating green pea aphids, *Myzus persicae*, with azadirachtin affects the predatory ability and protective enzyme activity of harlequin ladybirds, *Harmonia axyridis*. Ecotoxicol. Environ. Saf..

[31] Rondoni G., Ielo F., Ricci C., Conti E. (2017). Behavioural and physiological responses to prey-related cues reflect higher competitiveness of invasive vs. native ladybirds. Sci. Rep..

[32] Tian M., Xu L., Jiang J., Zhang S., Liu T., Xu Y. (2020). Host plant species of *Bemisia tabaci* affect orientational behavior of the ladybeetle *Serangium japonicum* and their implication for the biological control strategy of whiteflies. Insects.

[33] Camara Siqueira da Cunha J., Swoboda M.H., Sword G.A. (2022). Olfactometer responses of convergent lady beetles *Hippodamia convergens* (Coleoptera: Coccinellidae) to odor cues from aphid-infested cotton plants treated with plant-associated fungi. Insects.

[34] Hong M.W., Wang Y., Lu G. (2020). UV-Fenton degradation of diclofenac, sulpiride, sulfamethoxazole and sulfisomidine: Degradation mechanisms, transformation products, toxicity evolution and effect of real water matrix. Chemosphere.

[35] Zhu Q.Y., Yang Y., Lao Z.T., Zhong Y.Y., Zhang B.J., Cui X.P., O’Neill P., Hong D., Zhang K., Zhao S.Q. (2020). Synthesis, insecticidal activities and resistance in *Aedes albopictus* and cytotoxicity of novel dihaloacetylated heterocyclic pyrethroids. Pest. Manag. Sci..

[36] Moustafa S.A., Nabih S.A., Kenawy I.M., Abou-Elzahab M.M., Abdel-Mogib M. (2012). Clove oil as a source of attractant pheromones to the fruit flies, *Ceratits capitata* (Wiedemann) and *Bactrocera zonata* (saunders) (Dipetra: Tephritidae). J. Plant. Protect. Pathol..

[37] Irahal I.N., Guenaou I., Lahlou F.A., Hmimid F., Bourhim N. (2022). *Syzygium aromaticum* bud (clove) essential oil is a novel and safe aldose reductase inhibitor: In silico, in vitro, and in vivo evidence. Hormones.

[38] Bulawan J.A., Mpia L., Tojang D., Hasbiadi R. (2022). The effectiveness of various aromatic vegetable extracts to control fruit fly (*Bactrocera dorsalis*) pests in chili. Agrotech. J..

[39] Chaieb K., Hajlaoui H., Zmantar T., Kahla-Nakbi A.B., Rouabhia M., Mahdouani K., Bakhrouf A. (2007). The chemical composition and biological activity of clove essential oil, *Eugenia caryophyllata* (*Syzigium aromaticum* L. Myrtaceae): A short review. Phytother. Res..

[40] Gaire S., O’Connell M., Holguin F.O., Amatya A., Bundy S., Romero A. (2017). Insecticidal properties of essential oils and some of their constituents on the *Turkestan Cockroach* (Blattodea: Blattidae). J. Econ. Entomol..

[41] Pandiyan G.N., Mathew N., Munusamy S. (2019). Larvicidal activity of selected essential oil in synergized combinations against *Aedes aegypti*. Ecotoxicol. Environ. Saf..

[42] Ben Hassine D., Kammoun El Euch S., Rahmani R., Ghazouani N., Kane R., Abderrabba M., Bouajila J. (2021). Clove buds essential oil: The impact of grinding on the chemical composition and its biological activities involved in consumer’s health security. Biomed. Res. Int..

[43] Niogret J., Epsky N.D. (2018). Attraction of *Ceratitis capitata* (Diptera: Tephritidae) sterile males to essential oils: The importance of linalool. Environ. Entomol..

[44] Zhang X.G., Wei C.M., Miao J., Zhang X.J., Wei B., Dong W.X., Xiao C. (2019). Chemical compounds from female and male rectal pheromone glands of the guava fruit fly, *Bactrocera correcta*. Insects.

[45] Li H.J., Ren L., Xie M.X., Gao Y., He M.Y., Hassan B., Lu Y.Y., Cheng D.F. (2020). Egg-surface bacteria are indirectly associated with oviposition aversion in *Bactrocera dorsalis*. Curr. Biol..

[46] Ghabbari M., Guarino S., Caleca V., Saiano F., Sinacori M., Baser N., Mediouni-Ben Jemâa J., Lo Verde G. (2018). Behavior-modifying and insecticidal effects of plant extracts on adults of *Ceratitis capitata* (Wiedemann) (Diptera Tephritidae). J. Pest. Sci..

[47] Haro-González J.N., Castillo-Herrera G.A., Martínez-Velázquez M., Espinosa-Andrews H. (2021). Clove essential oil (*Syzygium aromaticum* L. Myrtaceae): Extraction, chemical composition, food applications, and essential bioactivity for human health. Molecules.

[48] Ikawati S., Himawan T., Abadi A.L., Tarno H. (2021). Toxicity nanoinsecticide based on clove essential oil against *Tribolium castaneum* (Herbst). J. Pestic. Sci..

[49] Parker R.A., Gabriel K.T., Graham K.D., Butts B.K., Cornelison C.T. (2022). Antifungal activity of select essential oils against *Candida auris* and their interactions with antifungal drugs. Pathogens.

[50] Ono H. (2022). Functional characterization of an olfactory receptor in the Oriental fruit fly, *Bactrocera dorsalis*, that responds to eugenol and isoeugenol. Comp. Biochem. Physiol. B.

[51] Pramod K., Ansari S.H., Ali J. (2010). Eugenol: A natural compound with versatile pharmacological actions. Nat. Prod. Commun..

[52] Arancibia M., Rabossi A., Bochicchio P.A., Moreno S., López-Caballero M.E., Gómez-Guillén M.D.C., Montero P. (2013). Biodegradable films containing clove or citronella essential oils against the Mediterranean fruit fly *Ceratitis capitata* (Diptera: Tephritidae). J. Agric. Food Technol..

[53] Fernandes M.J.G., Pereira R.B., Pereira D.M., Fortes A.G., Castanheira E.M.S., Gonçalves M.S.T. (2020). New eugenol derivatives with Wenhanced insecticidal activity. Int. J. Mol. Sci..

[54] FDA (2018). Direct Food Substances Affirmed as Generally Recognized as Safe.

[55] Raghu S., Clarke A.R., Yuval B. (2002). Investigation of the physiological consequences of feeding on methyl eugenol by *Bactrocera cacuminata* (Diptera: Tephritidae). Environ. Entomol..

[56] Pagadala Damodaram K.J., Kempraj V., Aurade R.M., Venkataramanappa R.K., Nandagopal B., Verghese A., Bruce T. (2014). Oviposition site-selection by *Bactrocera dorsalis* is mediated through an innate recognition template tuned to γ-octalactone. PLoS ONE.

[57] Gadenne C., Barrozo R.B., Anton S. (2016). Plasticity in insect olfaction: To smell or not to smell?. Annu. Rev. Entomol..

[58] Malacrida A.R., Gomulski L.M., Bonizzoni M., Bertin S., Gasperi G., Guglielmino C.R. (2007). Globalization and fruit fly invasion and expansion: The medfly paradigm. Genetica.

[59] Cruz R.C.D., Silva S.L.C.E., Souza I.A., Gualberto S.A., Carvalho K.S., Santos F.R., Carvalho M.G. (2017). Toxicological evaluation of essential oil from the leaves of *Croton argyrophyllus* (Euphorbiaceae) on *Aedes aegypti* (Diptera: Culicidae) and *Mus musculus* (Rodentia: Muridae). J. Med. Entomol..

[60] Li Y.J., Zhuang Y.L., Tian W.H., Sun L.P. (2020). In vivo acute and subacute toxicities of phenolic extract from rambutan (*Nephelium lappaceum*) peels by oral administration. Food Chem..

[61] He F., Sun S., Sun X., Ji S., Li X., Zhang J., Jiang X. (2018). Effects of insect growth-regulator insecticides on the immature stages of *Harmonia axyridis* (Coleoptera: Coccinellidae). Ecotoxicol. Environ. Saf..

[62] He F., Sun S., He L., Qin C., Li X., Zhang J., Jiang X. (2020). Responses of *Harmonia axyridis* (Coleoptera: Coccinellidae) to sulfoxaflor exposure. Ecotoxicol. Environ. Saf..

[63] Cheng Z., Wang D., Han S., Zuo C., He Y. (2022). Transcriptome analysis in the thiamethoxam resistance of seven-spot ladybird beetle, *Coccinella septempunctata* (Coleoptera: Coccinellidae). Ecotoxicol. Environ. Saf..

[64] Beers E.H., Mills N.J., Shearer P.W., Horton D.R., Milickzy E.R., Amarasekare K.G., Gontijo L.M. (2016). Nontarget effects of orchard pesticides on natural enemies: Lessons from the field and laboratory. Biol. Control..

[65] Toledo P.F.S., Viteri Jumbo L.O., Rezende S.M., Haddi K., Silva B.A., Mello T.S., Della Lucia T.M.C., Aguiar R.W.S., Smagghe G., Oliveira E.E. (2020). Disentangling the ecotoxicological selectivity of clove essential oil against aphids and non-target ladybeetles. Sci. Total. Environ..

[66] Yun X., Huang Q., Rao W., Xiao C., Zhang T., Mao Z., Wan Z. (2017). A comparative assessment of cytotoxicity of commonly used agricultural insecticides to human and insect cells. Ecotoxicol. Environ. Saf..

[67] Papoutsis I., Nikolaou P., Spiliopoulou C., Pistos C., Stefanidou M., Athanaselis S. (2012). A simple and sensitive GC/MS method for the determination of atropine during therapy of anticholinesterase poisoning in serum samples. Drug. Test. Anal..

[68] Miyazawa M., Hisama M. (2001). Suppression of chemical mutagen-induced SOS response by alkylphenols from clove (*Syzygium aromaticum*) in the *Salmonella typhimurium* TA1535/pSK1002 umu test. J. Agric. Food Chem..

[69] Nirmala M.J., Durai L., Gopakumar V., Nagarajan R. (2019). Anticancer and antibacterial effects of a clove bud essential oil-based nanoscale emulsion system. Int. J. Nanomed..

[70] Gooderham N.J., Cohen S.M., Eisenbrand G., Fukushima S., Guengerich F.P., Hecht S.S., Rietjens I.M.C.M., Rosol T.J., Davidsen J.M., Harman C.L. (2020). FEMA GRAS assessment of natural flavor complexes: Clove, cinnamon leaf and West Indian bay leaf-derived flavoring ingredients. Food Chem. Toxicol..

